# Commitment to Self-Tracking Among Wearable-Device Users: Validation of the C2ST Scale and Known-Groups Evidence

**DOI:** 10.3390/ejihpe16020026

**Published:** 2026-02-13

**Authors:** Jiri Remr

**Affiliations:** INESAN (Institute for Evaluations and Social Analyses), Sokolovská 351/25, 18600 Prague, Czech Republic; jiri.remr@inesan.eu

**Keywords:** self-tracking, commitment, psychometrics, self-tracker identity, quantified self, wearables, scale validation, confirmatory factor analysis

## Abstract

Background/Objectives: Commitment to self-tracking refers to the extent to which individuals are dedicated to the practice of wearable- and app-based self-monitoring. This commitment is behaviorally grounded and captures users’ sustained investment in wearable and app-based self-monitoring. The objective of this study was to validate the Commitment to Self-Tracking (C2ST) scale in Czechia by examining its dimensionality, confirmatory model fit, reliability, and known-groups evidence among self-tracking device users. Methods: The results were obtained from a face-to-face survey of a sample of 502 self-tracking device users who were recruited from the Czech general population using address-based sampling. The sample was randomly split into two subsamples for exploratory factor analysis (EFA) and confirmatory factor analysis (CFA). Item- and scale-level descriptive statistics and internal consistency (Cronbach’s α, McDonald’s ω) were calculated. The EFA was utilized to evaluate the factorability and latent structure of the model, and the CFA was employed to assess the model’s fit. The known-groups validity was examined using nonparametric group comparisons (Kruskal–Wallis H and Mann–Whitney U tests) with theoretically relevant external indicators, such as social comparison orientation, willingness to share data, perceived usefulness of tracking, and self-rated health. Results: The C2ST score demonstrated a full range of theoretical variation, exhibiting minimal floor (7.2%) and ceiling (2.0%) effects and a nearly symmetrical distribution. The internal consistency of the scale was found to be high (α = 0.968; ω = 0.968), and the corrected item-total correlations were uniformly high. The EFA supported a single-factor solution that explained 74.4% of the variance. The CFA model demonstrated a unidimensional structure, indicating that the observed variables were best explained by a single factor. An improved model attained an adequate-to-excellent fit (RMSEA = 0.051; SRMR = 0.018; CFI = 0.991; TLI = 0.986) and accounted for substantial item variance (R^2^ = 0.60–0.82). The known-groups validity was supported by pronounced differences in C2ST scores across social comparison and data-sharing orientations, as well as perceived usefulness of tracking for health and training goals (all *p* < 0.001). Conclusions: The Czech C2ST has been demonstrated to exhibit high reliability and a clear, unidimensional structure. Additionally, it exhibited robust CFA support and theory-consistent known-groups validity among self-tracking device users. The scale is appropriate for research on self-tracking commitment and persistence.

## 1. Introduction

Consumer self-tracking, enabled by wearable devices and companion apps that continuously capture, summarize, and visualize personal behavioral and physiological data, has become a mainstream form of everyday automation (Baumgart & Wiewiorra, 2016; Cusack et al., 2024; Lupton, 2014). Self-tracking is examined across disciplines as a mechanism that supports health and well-being, goal pursuit, and self-regulation. It has also become a prominent empirical domain within personal informatics and the “quantified self” literature (Feng et al., 2021). As this body of research has grown, a dual message has emerged from the literature reviews: self-tracking can yield meaningful benefits for some users and use cases, but outcomes depend on how tracking is embedded in everyday routines, how data are interpreted, and whether use is sustained over time (Feng et al., 2021; González-Baldovinos et al., 2025; Kersten-van Dijk et al., 2017).

A distinguishing characteristic of consumer self-tracking is its predominance outside of clinical settings, accompanied by a heterogeneity of objectives, variable levels of engagement, and fluctuations in the intensity of engagement (Panahi, 2025; Piwek et al., 2016). Conventional models delineate personal informatics as an iterative process, encompassing the stages of decision-making regarding data collection, data integration and processing, reflection, and subsequent action (I. Li et al., 2010). Recent research in the field of lived informatics has highlighted that real-world tracking is seldom linear. Conversely, users frequently encounter interruptions, transition between tools, and engage in occasional bursts of activity rather than consistent, uninterrupted monitoring (Rooksby et al., 2014). This variability complicates the development and application of theory and measurement because the same person using the same device may exhibit different tracking patterns due to changes in life circumstances, motivations, social contexts, and interpretations of feedback over time (Rooksby et al., 2014).

In accordance with these observations, the sustained engagement necessary for consumer self-tracking represents a significant challenge (Hermsen et al., 2017). Evidence of discontinuance and intermittent use has generated a marked increase in interest in the psychological and behavioral determinants of continued engagement. Researchers are also examining constructs that can distinguish superficial uptake from deeper integration and persistence. However, research frequently relies on simplified measures, such as device ownership, frequency of data checking, or number of recorded days. While these metrics are informative, they are typically insufficient in capturing the quality and meaning of a user’s relationship with tracking. This limitation mirrors broader debates in digital behavior change research, where engagement is increasingly conceptualized as a complex construct combining behavioral and experiential components, and where valid measurement remains methodologically demanding (Perski et al., 2020).

In light of these findings, Hancı et al. (2020) advanced the concept of commitment to self-tracking as a construct designed to foster a more comprehensive and behaviorally grounded relationship between the user and the practice. This construct goes beyond mere indices of frequency or device interaction. Conceptually, commitment is defined by three interrelated behavioral manifestations: The first component is a dedication to the tracker as a device, which manifests in continuous wearing and device-centered routines. The second component is personal investment in self-tracking as a functional activity, which involves investing time, cognitive effort, and resources in collecting and interpreting data. The third component is projected social identity, which includes sharing, community interaction, advocacy, and competitive or comparative behaviors. The authors conceptualize commitment as a single latent construct that is expressed across these behavioral domains (Hancı et al., 2021).

To operationalize this construct, Hancı et al. (2020) developed the Commitment to Self-Tracking Scale (C2ST), a brief self-report instrument based on behavior-focused indicators derived from desk research and engagement with quantified-self communities. In the initial validation, exploratory factor analysis supported a one-factor structure with high reliability (α = 0.91). The validity of the C2ST scale was demonstrated through its theoretically consistent associations with self-determination theory. Specifically, the C2ST scale correlated positively with autonomous motivation and negatively with controlled motivation. The authors posit that the C2ST scale is a valuable instrument for investigating the abandonment problem and for differentiating user groups and tracking patterns.

Notwithstanding these contributions, two gaps remain salient. Firstly, the psychometric evidence for the C2ST scale remains predominantly constrained to the scale’s original development context. Cumulative research and cross-study comparability necessitate the demonstration of scale measurement structure, reliability, and validity generalization across languages, sociocultural environments, and sampling frames. Secondly, even within the rapidly expanding body of literature on self-tracking, cross-national validation studies remain relatively scarce. This imposes limitations on the capacity for meaningful comparisons and may result in the privileging of constructs that are calibrated to particular cultural and technological environments (Feng et al., 2021; Teixeira et al., 2012). Addressing these gaps does not constitute a mere formal psychometric exercise. In order for commitment to serve as a theoretically informative predictor of sustained engagement and related outcomes, it must be measured in ways that are comparable across the various contexts in which self-tracking is practiced (Neff, 2003b).

The present study validated the C2ST scale in a new environment by focusing on self-tracking device users in Czechia. The Czech context is pertinent for two reasons. Firstly, self-tracking practices have been shown to support everyday health management and lifestyle optimization (Lyall & Robards, 2018; Pantzar & Ruckenstein, 2017). Secondly, the adoption of a behavior-based commitment measure offers a rigorous examination of conceptual transferability. The manifestation of tracking, particularly norms concerning sharing, community engagement, and competitive comparison, may exhibit cultural patterns, even when the underlying construct remains stable (Al Khaldy et al., 2025; Paré et al., 2018).

Czechia provides a high-connectivity context with widespread use of digital technologies. Eurostat indicators (Eurostat, 2025) show that 95% of residents aged 16–74 used the internet in the past three months in 2025. Moreover, the use of smartphones was prevalent across all adult age groups, and it reached 82%, and on top of that, 70% of individuals aged 16–74 reported at least basic digital skills. In the domain of health, 73% of individuals aged 16–74 reported using the internet to seek health-related information in 2025. However, the proliferation of connected wearables is not uniform; data indicate that 44% of the population used an internet-connected wearable device, e.g., smartwatch or fitness band in 2024 (Eurostat, 2025).

Accordingly, this study aimed to validate the Czech C2ST by assessing reliability, factor structure (EFA/CFA), and known-groups validity among self-tracking device users. The study addressed the following research questions:

1. Does the Czech C2ST replicate the original one-dimensional measurement model?
2. Does the Czech version demonstrate psychometric properties adequate for use among self-tracking device users in this setting?
3. Does the scale show sufficient sensitivity to capture differences in commitment across relevant user segments?

## 2. Materials and Methods

### 2.1. Study Design

The survey employed an address-based household sampling approach using a census-derived address frame of private dwellings, which is regarded as the most accurate and complete sampling frame available. In the first step, the territorial units (fieldwork sampling points) were defined as small geographic areas for performing the address selection aiming to ensure national coverage across all settlement-size strata; within sampling points, addresses were selected systematically from the sampling frame. In the second step, in each dwelling unit that was contacted, the interviewers enumerated all eligible household members and selected one respondent using a standardized within-household randomization rule based on Kish table. At this stage, the usual inclusion criteria were applied; individuals must be residing in the given household, must be 15–74 years of age, and must be capable of completing a Czech-language interview. The third step involved the screening of respondents for the use of self-tracking technologies (i.e., smartwatch or fitness band), or mobile applications for self-tracking. Those respondents who satisfied the device-use criterion were then invited to complete the C2ST module, thus forming the self-tracking device user sample for the study.

### 2.2. Participants and Procedures

As part of the second step, 1982 people were asked to participate in face-to-face interviews during May 2025, and 1053 agreed to participate in the study, leading to the response rate of 53.1% (calculated in accordance with AAPOR-5 convention). However, since some interviews were incomplete, the sample included 1046 respondents, who represented the general population. Missing cases were handled by listwise exclusion, such that psychometrics analyses were conducted on complete cases only. Of these 1046 respondents, 502 were identified in the third step as users of self-tracking devices, and this group served as an analytical dataset.

The sample size planning was conducted in accordance with the established recommendations for factor-analytic validation and reliability assessment (Anthoine et al., 2014; Lorenzo-Seva & Ferrando, 2024). The objective was to obtain a minimum of 480 device users that would permit splitting the self-tracking device user sample into two halves and performing the EFA and CFA on each subsample independently. This was important to strengthen the internal validity of the study and reduce reliance on chance. The gross number of contacted individuals was set to achieve this self-tracking device users target under anticipated response and screening yields, within the logistical constraints of fieldwork. The achieved self-tracking device user sample of 502 respondents, i.e., 251 cases in each subsample, meets the aforementioned requirements and supports robust estimation for internal consistency, exploratory structure detection, and confirmatory model testing. Sample characteristics (gender, age group, size of settlement, and education) are reported in Table 1 for each subsample and for the total sample, together with Czech population parameters to contextualize the specifics of the self-tracking device users. Population parameters are provided only for contextual comparison; the analyses were performed of the sample that represents screened current self-tracking device users. No post-stratification weights were applied.

All respondents participated voluntarily and provided informed consent prior to initiating the interview. The confidentiality of their responses was assured. The data underwent processing and storage in an anonymized form, and analyses were conducted on de-identified records. The fieldwork procedures were carried out in accordance with the ethical principles outlined in the Helsinki Declaration (World Medical Association, 2025). Prior to data collection, ethical approval was obtained from the INESAN Ethical Committee (IREBA/2025/423, 23 April 2025).

### 2.3. Translation and Adaptation of the Scale

The original C2ST scale is a 12-item, behavior-based self-report instrument designed to capture commitment to self-tracking through behaviors reflecting device integration, functional investment in tracking as an activity, and socially projected self-tracker identity. Items used a 7-point Likert-type response format in that 1 was “strongly disagree” and 7 was “strongly agree”.

To ensure the linguistic and conceptual equivalence of the Czech C2ST scale, the instrument was translated and adapted using a multi-step procedure consistent with the best-established practices for cross-cultural scale adaptation (Beaton et al., 2000; Sousa & Rojjanasrirat, 2011). Briefly, two forward translations from English to Czech were prepared by bilingual translators, and discrepancies were reconciled through expert review focused on semantic, idiomatic, and conceptual equivalence. Next, a back-translation from Czech to English was produced by an independent translator who was unfamiliar with the original wording. Divergences were then reviewed and resolved to preserve the functional meaning (Gjersing et al., 2010; Behr, 2017). Then, the draft Czech version was pilot-tested using cognitive interviewing with 19 Czech respondents of varying ages and levels of education to identify ambiguities, specific interpretations, and problematic wording (Van Teijlingen & Hundley, 2010). Minor revisions, consisting of wording refinements, were implemented in three items where comprehension issues were observed. While maintaining the original intent of the items and their behavioral framing, refinements included the substitution of less common expressions with everyday equivalents and the resolution of ambiguous phrasing. These edits were reviewed by the translation team to ensure semantic equivalence. The number of items, the response format, and the behavioral framing of the construct remained constant.

### 2.4. Measures

#### 2.4.1. Commitment to Self-Tracking (C2ST)

The Czech C2ST scale included the same 12 items as the original C2ST scale, which covered device-centered routines (e.g., going back to retrieve the device), continuous wear (i.e., sleeping with the device), informational and self-regulatory engagement (for instance, analyzing tracked behavior and updating goals), monetary and material investment (especially, willingness to pay extra and purchasing accessories), and socially projected engagement including data sharing, tracking recommendation, or interacting with tracking communities. A total score was computed by summing the scores of each case, with higher scores indicating stronger commitment.

#### 2.4.2. Auxiliary Indicators for Known-Groups Validity

To assess the known-groups validity of C2ST scores, we examined whether they differed systematically across a set of external indicators theoretically connected to sustained engagement in self-tracking. First, respondents reported their self-rated health status using a standard item widely used in population health research (Bombak, 2013). Five-categories self-rated health was collapsed to 3 groups to ensure sufficient cell sizes (sensitivity analyses using the original 5-category variable yielded comparable conclusions). Additionally, participants provided a brief assessment of their self-rated physical status, which was operationalized as a dichotomous indicator (good versus bad).

Because commitment to self-tracking is expected to be reflected in individual routines, social orientations, and motivational factors (Lupton, 2016; Choe et al., 2014; Gimpel et al., 2013), we included two additional indicators: respondents’ agreement with statements expressing a desire to compare their results with others’ and to share their self-tracking data. Both of these indicators were re-coded into three categories, which are agree, neither/nor, and disagree. We also measured an affective correlate of tracking by asking whether respondents felt less anxious about their health in relation to self-tracking (agree; neither nor; disagree), reflecting the notion that tracking may shape health-related emotions and perceived reassurance or concern (Joshi et al., 2025; Patel et al., 2015; Ruckenstein & Pantzar, 2017).

To capture the instrumental dimension of tracking, we assessed the perceived usefulness of self-tracking for health- and performance-related goals (Stiglbauer et al., 2019; Simpson & Mazzeo, 2017; Sharon & Zandbergen, 2017). Specifically, participants indicated whether they considered self-tracking useful for four domains: monitoring changes in health, getting rid of a health-related bad habit, motivating themselves to achieve health goals, and tracking progress in sports/training. Finally, because commitment plausibly correlates with the intensity of behavioral use (Sheeran & Webb, 2016; Silverman & Barasch, 2016), we included the frequency of device use as an additional behavioral indicator. This variable was categorized as follows: several times a day/constantly, once a day, several times a week, or less often. These auxiliary variables enabled us to test whether higher commitment, as captured by C2ST scale, is associated with more socially embedded, instrumentally valued, and behaviorally intensive forms of self-tracking.

Because some auxiliary indicators (e.g., particularly social comparison orientation and willingness to share tracking data) capture instrumental appraisals closely related to commitment behaviors, results are interpreted as proximal known-groups evidence rather than independent criterion validity. More distal auxiliary indicators, such as self-rated health, tracking-related anxiety reduction, perceived instrumental usefulness, and behavioral usage frequency, have been demonstrated to help mitigate this concern.

### 2.5. Data Analysis

Descriptive statistics were computed for each item and for the total C2ST score, including means, standard deviations, and distributional diagnostics. The assessment of floor and ceiling effects was conducted by determining the proportion of respondents who scored at the theoretical minimum or maximum, respectively (Ho & Yu, 2015; Šimkovic & Träuble, 2019; Cain et al., 2017). The internal consistency of the scale was evaluated using Cronbach’s α and McDonald’s ω (Hayes & Coutts, 2020; Taber, 2018; Kalkbrenner, 2023). Furthermore, to summarize the quality of measurement of the latent construct at the factor level composite reliability (CR) and average variance extracted (AVE) were used (Fornell & Larcker, 1981; Remr, 2023).

Given the nature of the C2ST items as ordinal Likert-type responses, the interitem associations were summarized using Kendall’s tau-b, a statistical method that is particularly well-suited for monotonic associations within the context of ordinal measurement. This approach is further advantageous in that it explicitly accounts for the presence of tied ranks, which are a common occurrence in Likert data (Field, 2017; Yaska & Nuhu, 2024).

An exploratory factor analysis (EFA) was conducted on the first random subsample (n = 251) to examine the underlying dimensionality with a common-factor extraction method, namely principal axis factoring (Fabrigar & Wegener, 2012). The sampling adequacy and factorability were evaluated using the Kaiser–Meyer–Olkin (KMO) measure and Bartlett’s test of sphericity (Shrestha, 2021; Pett et al., 2003). The number of factors retained was determined using standard criteria, such as eigenvalues and scree/interpretability, as well as the theoretical expectation of a unidimensional commitment construct (Loewen & Gonulal, 2015; Hogarty et al., 2005). Because a single factor was retained, rotation was not applied.

A confirmatory factor analysis (CFA) was performed on a second subsample (n = 251) to verify the factor structure suggested by EFA (C. H. Li, 2016). The model was estimated using the maximum likelihood (ML) method methodology that has been demonstrated to be viable for 7-point items. To substantiate this selection, an examination of univariate item distributions (skewness and kurtosis) and multivariate normality diagnostics was conducted. The substantive conclusions, especially, the factor structure and item-factor relations were then compared across estimators. Moreover, given ordinal nature of the items, bootstrap standard errors and bias-corrected confidence intervals were employed, with 2000 replications. The model’s fit was evaluated using a combination of absolute and incremental indices including the Bollen–Stine bootstrap test, the root mean square error of approximation (RMSEA), the standardized root mean square residual (SRMR), the goodness of fit index (GFI), the comparative fit index (CFI), the Tucker–Lewis index (TLI), and the normed fit index (NFI). In accordance with prevailing guidelines (Hu & Bentler, 1999), we interpreted CFI/TLI values of 0.90–0.95 and RMSEA/SRMR values of 0.08 or less (ideally 0.06 or less) as indicative of an acceptable to good fit (West et al., 2012). An inspection of the modification indices (MI) was conducted to ascertain the presence of localized dependencies among conceptually overlapping items. Residual covariances were only released when they were theoretically interpretable, i.e., when they occurred in a shared micro-routine, near-synonymous wording, or overlapping context within the same behavioral manifestation. Furthermore, this release of residual covariances did not alter the substantive meaning of the general factor.

The known-groups validity was assessed by testing the hypothesis that C2ST scores would differ across the external indicators previously described. Due to the ordinal nature of the outcomes and the unequal group sizes in several indicators, the evaluation of group differences was conducted using nonparametric tests. The Kruskal–Wallis H test was employed for three-category variables, while the Mann–Whitney U test was utilized for binary variables. The significance of these results was assessed at conventional levels of statistical significance (*p* < 0.05 and *p* < 0.01). In the context of known-groups comparisons, we also report effect sizes in conjunction with *p*-values. For Kruskal–Wallis tests, epsilon-squared (ε^2^) is reported to quantify the proportion of rank variance attributable to group membership. For Mann–Whitney tests, the Cliff’s delta (δ) is reported as an effect size.

All analyses were performed with IBM SPSS Statistics 28 (IBM Corp., Armonk, NY, USA) and AMOS 24.

## 3. Results

Table 1 summarizes the demographic composition of the two subsamples and illustrates how the total sample of self-tracking device users (n = 502) compares to the general population of Czechia. In terms of gender, the self-tracking device user sample did not differ significantly from the general population (47.8% males and 52.2% females). However, self-tracking device users differ from the general population with respect to age and size of settlement. The self-tracking device user sample is skewed toward younger respondents, with a higher proportion of individuals aged 15–29 years (30.9% vs. 20.1% in the general population) and a lower proportion of individuals aged 60–74 years (17.2% vs. 22.9%). The self-tracking device user sample is also more urban, with a higher proportion of individuals living in settlements with more than 100,000 inhabitants (25.0% vs. 22.7%) and a lower proportion of individuals living in small municipalities with fewer than 1000 inhabitants (9.7% vs. 16.9%). Additionally, the educational profile showed a lower percentage of respondents with elementary education (8.4% vs. 14.0%) and a higher percentage with secondary education (44.4% vs. 34.5%) than the general population. Given the study’s focus on scale validation, it is important to examine how similar the structures of both subsamples are. In this respect, Table 1 shows that the EFA and CFA subsamples are comparable across all measures statistics, i.e., gender, age, size of settlement, and education, supporting the appropriateness of the data.

Item-level descriptive statistics for the C2ST scale are reported in Table 2. Across the 12 items, average item ratings were around the mid-range, suggesting that the sample largely comprised moderately committed self-trackers rather than uniformly high-commitment power users. The lowest mean was observed for Item D (“I share my data with others”), with M = 3.34, whereas the highest mean was observed for Item E (“I make extra effort to reach my goal”), with M = 4.19. The means were accompanied by substantial variability, with item standard deviations ranging from SD = 1.909 to SD = 2.239. The observed dispersion suggests adequate variability for factor-analytic modeling, as it indicates that items are not suffering from strong response clustering.

**Figure 1:**
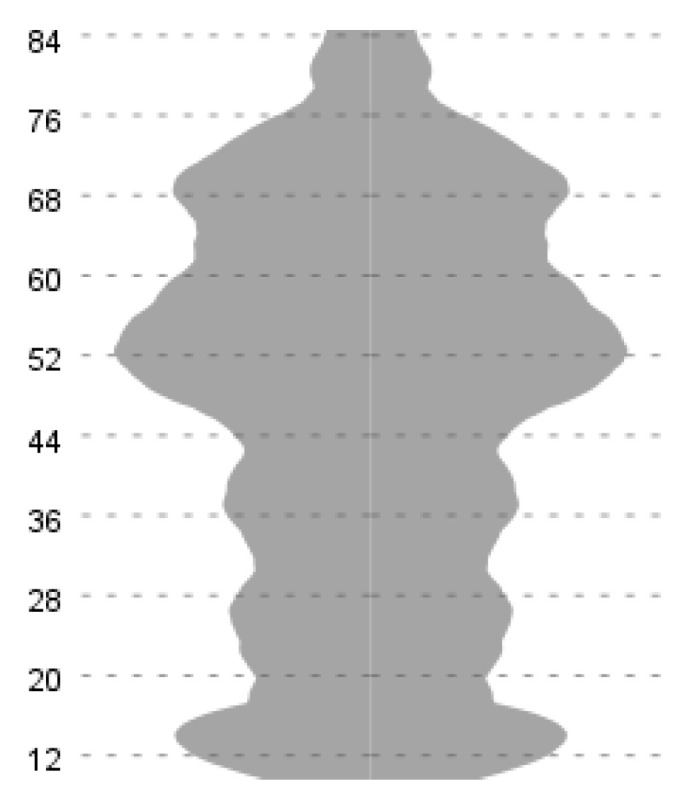
C2ST Scale Distribution. Note: Minimum = 12, Maximum = 84, Mean = 46.17, SD = 20.312.

At the scale level, the summed C2ST score had a mean of 46.17, a standard deviation of 20.31, and a median of 50. A preliminary analysis of the distributional indices indicated that the scores exhibited approximate symmetry (skewness = −0.167) and relatively flat tails (kurtosis = −1.038). As demonstrated in Figure 1, the distribution encompassed the entire theoretical range of the instrument, ranging from a minimum score of 12 to a maximum score of 84. The impact of floor and ceiling effects was negligible, with 7.2% of respondents attaining the theoretical minimum and 2.0% achieving the theoretical maximum. The results indicate that the C2ST scale effectively differentiates between levels of commitment, without converging toward a single extreme. This is an important prerequisite for robust structural testing and subsequent validity analyses.

The reliability indicators further corroborated the measurement quality of the C2ST scale. The internal consistency of the scale was found to be high, with Cronbach’s alpha and McDonald’s omega both measuring 0.968. The corrected item demonstrated in Table 2 consistently elevated total correlations (0.804–0.869), thereby signifying the robust alignment of each item with the overall scale. The “α if deleted” scale demonstrated minimal change (0.965–0.966), indicating that no particular item significantly compromised the scale’s integrity and that each item contributed to an overall coherent construct.

Using Kendall’s tau-b, the inter-item correlations presented in Table A1 were consistently moderate to strong and statistically significant (*p* < 0.01), ranging from 0.523 to 0.708. This indicates a dense positive correlation structure, in which all items meaningfully relate to each other. However, they were not extreme enough to suggest duplicated items or problematic multicollinearity.

As Table 3 shows, EFA revealed a clear dominant single dimension. The factorability was adequate, as indicated by a KMO value of 0.960 and a highly significant Bartlett’s test of sphericity (χ^2^ = 3171.088, df = 66, *p* < 0.001). The first factor extracted by principal axis factoring (PAF) had an eigenvalue of 8.930 and explained 74.4% of the total variance. Item loadings were high, ranging from 0.817 to 0.885, as were communalities (0.668–0.783). This indicates that each item shares a substantial proportion of its variance with the latent construct. Measurement quality at the factor level was reinforced by an average variance extracted (AVE) of 0.72 and a composite reliability (CR) of 0.97, both of which reflect strong convergence of the indicators on the underlying factor.

CFA tested the one-factor structure more stringently. As shown in Table 4, the initial CFA specification yielded mixed evidence of fit. The RMSEA was 0.101, suggesting misfit, while the GFI was 0.881. The SRMR was 0.0284, and the incremental indices were acceptable to good (CFI = 0.957, TLI = 0.947, and NFI = 0.941). This configuration, i.e., strong incremental fit combined with weaker absolute fit, is often indicative of localized misfit rather than an incorrect global factor structure. Accordingly, a limited number of correlated residuals were introduced when supported by modification diagnostics and conceptually plausible due to overlapping content (i.e., closely related behavioral routines within the broader commitment construct). The resulting improved one-factor model showed good fit across indices (Table 4): RMSEA = 0.050, SRMR = 0.0177, GFI = 0.950, CFI = 0.990, TLI = 0.986, and NFI = 0.975. The Bollen–Stine bootstrap *p*-value improved from *p* < 0.001 in the initial model to *p* = 0.173 in the improved model.

**Figure 2:**
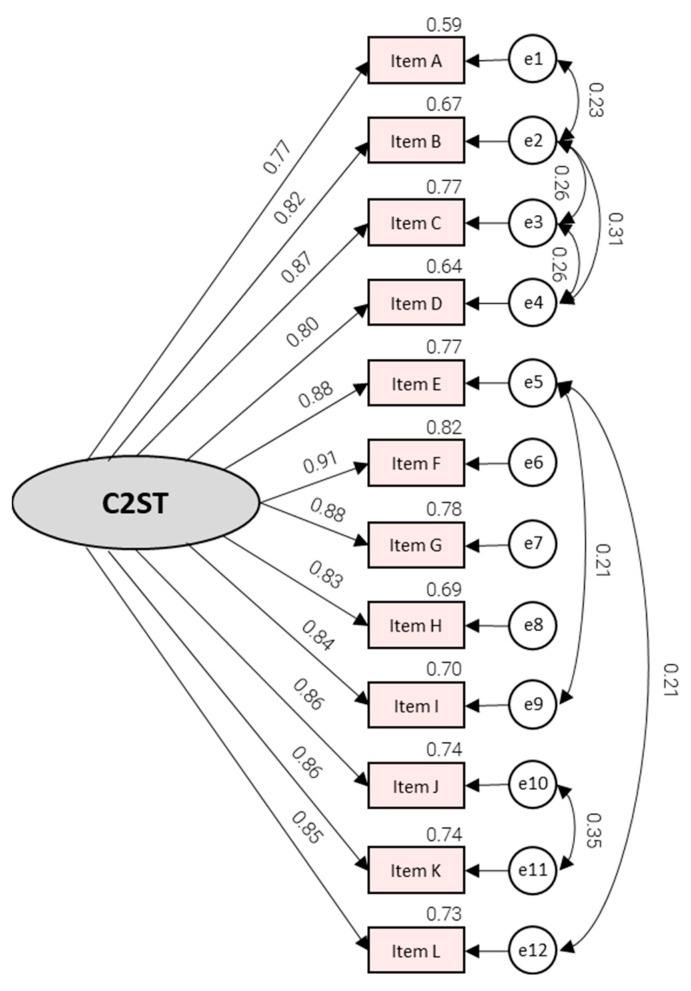
Confirmatory Factor Analysis (C2ST Scale—Improved model).

Figure 2 illustrates the strength and consistency of item–factor relations in the improved CFA model. Standardized factor loadings were uniformly high, ranging from λ = 0.77 for Item A to λ = 0.91 for Item F. The corresponding explained item variance was substantial with Item A at the lower end (R^2^ = 0.59) and Item F at the upper end (R^2^ = 0.82). The inclusion of correlated residuals among seven pairs of items suggests modest content-related dependencies layered on top of the dominant factor, which is consistent with the notion that some items capture similar routines within self-tracking behavior. Specifically, we freed the residual covariances between items A-B, B-C, C-D, B-D, E-I, J-K, and E-L. Freed residual covariances were limited to pairs with clear shared behavioral micro-routines or shared social-engagement content, suggesting method overlap beyond the general commitment factor.

Known-groups validity was evaluated by examining whether C2ST scores differed across external indicators that theory would predict to be related to sustained self-tracking (Table 5). For self-rated health status, C2ST scores differed significantly across groups (Kruskal–Wallis H = 21.477, *p* < 0.001). Respondents reporting good health had the highest C2ST scores (M = 49.05, SD = 19.40), those in the middle category (neither/nor) scored lower (M = 37.46, SD = 21.24), and respondents reporting bad health had the lowest scores (M = 28.38, SD = 13.96). This pattern indicates that commitment is systematically patterned across subjective health states.

The strongest evidence from known groups emerged for indicators that directly reflect social and behavioral engagement with tracking. C2ST scores varied according to the desire to compare results with others (H = 96.894, *p* < 0.001). Respondents who agreed with this orientation demonstrated a high level of commitment (M = 61.97, SD = 13.09). Those who were neutral demonstrated a moderate level (M = 48.98, SD = 16.60) and those who disagreed had lower C2ST score (M = 34.26, SD = 17.36). A similar pattern emerged for perceived reassurance-related effects. Respondents who agreed that tracking made them feel less anxious about their health scored higher (M = 55.70, SD = 18.26) than those who were neutral (M = 43.62, SD = 16.69) or disagreed (M = 38.22, SD = 21.03). Again, there was a strong overall difference (H = 33.715, *p* < 0.001).

C2ST scores were also tied to the perceived usefulness of tracking for specific goals. Respondents who found tracking health changes useful reported higher commitment (M = 52.21, SD = 18.49) than those who found it useless (M = 32.96, SD = 17.52); this difference was statistically significant (U = 2813.5, *p* < 0.001). The same pattern emerged when tracking was framed as useful for eliminating bad habits (useful: M = 54.19; not useful: M = 37.44; U = 3836.5, *p* < 0.001) or for motivating oneself to achieve health goals (useful: M = 54.50; not useful: M = 34.53; U = 3169.0, *p* < 0.001).

## 4. Discussion

The primary objective of this study was to validate the C2ST scale with a new population. A comprehensive study was conducted on a sample of Czech self-tracking device users. The findings indicate that the C2ST scale is a reliable and coherent measure of commitment to self-tracking. The scale exhibited a clear unidimensional structure, high internal consistency, and theoretically consistent associations with external indicators of sustained engagement. These results provide support for the notion that commitment is closely associated with instrumental and self-regulatory appraisals of self-tracking. It has been demonstrated that users who find tracking useful for the purposes of monitoring, motivation, and behavior change tend to exhibit the highest behavioral commitment, as measured by the C2ST scale. These results are consistent with the initial development of the scale, which conceptualized commitment to self-tracking as a single latent construct based on observable behavioral manifestations. The scale was developed as a standardized instrument to assess the extent to which users incorporate self-tracking into their daily lives.

The findings of this study demonstrate a high degree of convergence with the original C2ST development study, which reported a predominantly one-factor structure (eigenvalue = 6.5) with generally strong loadings and high internal consistency (α = 0.91) in a sample of wearable users. In the present validation, the factor solution was found to be even more saturated (eigenvalue = 8.93; 74.4% explained variance; uniformly high loadings), and internal consistency was very high (α = 0.968). These findings provide substantial support for the interpretation of commitment to self-tracking as a coherent latent construct expressed through tightly connected behavioral routines.

A central contribution of the present validation is the unusually strong support for unidimensionality. In the exploratory phase, the scale exhibited excellent factorability (KMO = 0.960; Bartlett *p* < 0.001) and produced a dominant single factor (eigenvalue = 8.93), which explained 74.4% of the variance with high loadings (0.84–0.89) and communalities. These results replicate the original validation, which also rejected a two-factor alternative and retained a one-factor solution with high reliability (α = 0.91). One plausible interpretation is that within a population of active self-tracking device users, the behavioral manifestations targeted by the C2ST scale (i.e., device integration, functional investment, and socially projected identity practices) tend to co-occur and reinforce each other, producing a strong general commitment factor.

The descriptive profile of the C2ST scale suggests that the instrument is sensitive to differentiate meaningful levels of commitment. The scores obtained fell across the entire theoretical range (12–84), indicating that the scale does not merely categorize the majority of self-tracking device users as either highly or minimally committed. This finding suggests that the scale is capable of capturing a nuanced range of user engagement levels. This distributional breadth is a critical component of psychometric evaluation, as factor-analytic stability and validity assessments necessitate sufficient variance. Concurrently, the scale demonstrated substantial internal consistency (0.968), and the corrected item-total correlations were robust. From a measurement perspective, these indicators imply that the scale did not introduce poorly functioning or ambiguous items. In essence, these findings suggest that commitment, as measured by the C2ST scale, is not merely a random assortment of unrelated habits but rather an interconnected set of practices.

CFA provided a more stringent test of structural validity and helped clarify the nature of residual model misfit. The initial one-factor CFA showed mixed absolute fit, while incremental indices and SRMR were acceptable to good. Allowing a limited set of residual covariances produced a substantially improved model with good fit. Meanwhile, standardized loadings remained high, and the explained item variance was substantial (R^2^ = 0.60–0.82). Although the chi-square statistic remained significant, the clear improvement in chi-square divided by degrees of freedom (from 3.55 to 1.65) supports the practical adequacy of the improved model.

Occurrence of correlated residuals should be interpreted cautiously; however, it does not undermine the unidimensionality. The original C2ST conceptualization explicitly describes commitment as behaviorally expressed through three defining elements: device integration, functional investment in tracking activity, and socially projected self-tracker identity. These are framed as manifestations of a single underlying disposition. Within this theoretical framework, local dependence between specific items is plausible, particularly when some indicators share similar situational contexts, micro-routines, or wording (e.g., closely related social expression behaviors, such as sharing, recommending, or engaging with tracking communities). The observed residual covariances may therefore reflect additional shared specificity within subsets of behaviors nested under a dominant general commitment factor. Replication is needed to confirm the stability of the covariances before treating the refined model as a standard scoring or measurement template. In this framework, the improved CFA model is not evidence of multidimensionality, but rather a practically realistic representation of a largely unidimensional construct with minor, interpretable departures from strict local independence.

Beyond the examination of internal structure, the Czech validation yielded substantively meaningful known-groups evidence. A significant divergence in C2ST scores was observed across indicators, which closely reflected the construct’s content and theoretical implications. A significant increase in commitment was observed among respondents who endorsed social comparison and sharing orientations, as well as among those who perceived tracking as beneficial for goal-related functions, such as health monitoring, habit modification, and goal pursuit motivation. The theoretical coherence of these associations stems from their ability to link commitment to the functional-investment component of self-tracking and the socially projected identity element, as outlined in the original framework. Furthermore, the elevated C2ST scores among respondents who reported diminished health-related anxiety through tracking suggest a potential correlation between commitment and mechanisms of reassurance and feedback that support sustained engagement. This interpretation aligns with the notion that tracking attains psychological significance and becomes integrated into daily self-regulation when it is sustained (Gimpel et al., 2013; Gilmore, 2016; Jones et al., 2022).

C2ST scores demonstrated a systematic correlation with self-rated health, with respondents reporting higher levels of commitment exhibiting improved health outcomes. This gradient is compatible with multiple non-exclusive explanations. First, it may reflect selection effects, whereby individuals with better functioning or a stronger capacity for routine maintenance are more likely to adopt and sustain self-tracking practices (Hermsen et al., 2017; Lupton, 2016). It may also reflect reinforcement processes, whereby ongoing tracking supports goal setting, feedback, and behavioral routines that occur alongside better perceived health (Pantzar & Ruckenstein, 2017; Izu et al., 2024). However, given the cross-sectional design, these associations should be interpreted as evidence of correlational validity rather than as causal effects of self-tracking on health.

The obtained results expand upon the initial validation by demonstrating the C2ST’s psychometric properties’ capacity for transfer across diverse social contexts. While the initial study primarily anchored known-groups validity in motivational profiles (positive associations with autonomous motivation and negative associations with controlled motivation) (Hancı et al., 2020), the present study provides complementary evidence through perceived utility, socially embedded engagement (e.g., social comparison and sharing), and reassurance-related experiences. This triangulation is valuable because it supports the construct’s broader nomological network from multiple angles and strengthens the interpretation of the C2ST scale as a behaviorally grounded measure that captures more than simple attitudinal endorsement (Neff, 2003a; Baumgart, 2017).

This study makes five major contributions. Firstly, it extends psychometric evidence for the C2ST scale beyond the original development context by demonstrating a stable unidimensional structure and high reliability. Secondly, the validation of the scale in a screened sample of self-tracking users drawn from a general-population sample serves to strengthen confidence in the scale’s portability across sampling frames and data-collection modes. Thirdly, the observed patterns of known-groups validity lend support to the interpretation of C2ST scores as meaningful markers of sustained engagement. Fourthly, the utilization of the C2ST scale is augmented, thereby expanding the range of applications. Fifthly, the text disseminates information regarding the commitment to self-tracking in Czechia. To the best of our knowledge, this constitutes the inaugural instance in which such information has been made available.

From an applied perspective, the validated C2ST scale presents numerous opportunities for research and practice. A robust measure of commitment can help advance the field beyond coarse proxies, such as ownership or usage frequency, which are informative but often insufficient for capturing the depth and meaning of self-tracking engagement. In health behavior change and personal informatics research, the C2ST scale can be used for theoretically informed segmentation, modeling sustained engagement and adherence, and investigating discontinuance trajectories. This is of particular importance in light of the recurrent evidence of diminishing utilization over time. Within the Czech context, the scale facilitates the examination of factors that contribute to the persistence of individuals in a given context, in contrast to those who disengage. It also allows for the investigation of the relationship between commitment and perceived benefits, as well as the anxieties that may impede or facilitate engagement. Additionally, the scale enables the analysis of how platform design and community features influence the promotion or hindrance of sustained engagement.

Several considerations frame interpretation and point to priorities for future research. First, the scale’s high reliability and strong factor saturation raise the possibility of partial item redundancy. Future work could evaluate whether a shorter form retains sufficient construct coverage while reducing respondent burden, especially in multi-construct survey batteries. In subsequent studies, a comparison of the one-factor solution with plausible alternatives, such as bi- or three-factor models, may be conducted to ensure that unidimensionality is not driven by shared method variance. Second, although including correlated residuals substantially improved the fit of the CFA model and can be theoretically justified, subsequent studies should replicate these model modifications and test whether the same specification holds in independent samples. These studies should also assess measurement invariance across key subgroups (e.g., gender and age). Third, known-groups validity was evaluated using cross-sectional group differences. Expanding the validation to include additional theoretically linked constructs (including the motivational profiles used in the original study) and predictive validity outcomes (e.g., sustained use and discontinuance over time) would strengthen the scale’s practical value. Finally, while the sample was gender-balanced, it was younger, more urban, and more educated than the general population, reflecting the differential adoption of wearable self-tracking technologies.

It is noteworthy that the psychometric analyses were conducted on a screened subsample of current self-tracking users. Consequently, the present results should be interpreted as evidence that the Czech C2ST scale functions reliably and coherently within active self-tracking device users. Accordingly, the present validation is best interpreted as applying to Czech self-tracking device users rather than providing normative benchmarks for the entire adult population. Furthermore, the presence of differences in magnitude can be attributed to the sampling frame and data collection method. It is possible that participants comprising the analyzed sample may have more stable tracking routines than other samples obtained, e.g., online. On top of that, the collection of data was conducted through in-person interactions, a method that might elicit social desirability responses, particularly in contexts involving advocacy or social display behaviors, such as the promotion of tracking, data sharing, and community interaction. Despite the emphasis on confidentiality and neutrality by interviewers, the possibility of such bias in the endorsement of expected behaviors cannot be discounted. Future studies could replicate the validation using self-administered modes (like, e.g., CAWI) and/or incorporate objective usage indicators to triangulate self-report. Finally, the proximity of auxiliary indicators and the potential for criterion contamination are salient issues. Therefore, extension of validation with other distal constructs, including autonomous/controlled motivation, habit strength, self-efficacy, and health anxiety, as well as objective tracking logs is recommended.

The present findings provide substantial evidence in support of the Czech C2ST scale. This scale has been demonstrated to be a psychometrically sound instrument, characterized by a stable unidimensional structure, strong reliability, and theory-consistent patterns. These results may serve as a foundational basis for subsequent studies that utilize commitment as an explanatory construct in the domains of self-tracking engagement, persistence, and long-term routine practices.

## 5. Conclusions

The present study validated the C2ST scale among Czech users of self-tracking devices in a novel environment. The results provide evidence that the Czech C2ST scale is a psychometrically robust, unidimensional instrument suitable for research on self-tracking engagement. The scale demonstrated significant variability in scores, with minimal floor and ceiling effects, and adequate sensitivity to differentiate between levels of commitment. The internal consistency was deemed to be very good.

The structural validity of the model was established in two stages: The EFA indicated a clear single-factor solution, and the CFA in an independent subsample corroborated the one-factor model. These findings support the interpretation of C2ST as a general commitment construct expressed through closely related behavioral routines of device integration, invested tracking practices, and socially oriented tracking behaviors. The known-groups validity of the C2ST scores was evident in two ways. First, the C2ST scores were significantly higher among respondents who endorsed a data-sharing orientation. Second, the C2ST scores were significantly higher among respondents who perceived self-tracking as useful for monitoring health changes. These associations align with the construct’s definition and strengthen the C2ST’s validity in the Czech context. However, generalizations to the broader population should be made with caution, given that the dataset consisted of current self-tracking device/app users identified through screening.

In practice, the Czech C2ST scale, which has been validated, provides researchers with a brief, behaviorally grounded tool that surpasses coarse proxies, such as device ownership or simple usage frequency. Future research should replicate the CFA specification in other independent samples, test invariance, examine predictive validity, and evaluate whether a short form can retain coverage while reducing the number of items.

## Figures and Tables

**Table 1:** Selected socio-demographic characteristics.

Variables		Total Population	EFA Sample	CFA Sample	Total Sample
Gender	Male	50.0%	45.4%	50.2%	47.8%
	Female	50.0%	54.6%	49.8%	52.2%
	Total	100.0%	100.0%	100.0%	100.0%
Age	15–29 years	20.1%	32.3%	29.5%	30.9%
	30–39 years	17.8%	19.6%	17.2%	18.4%
	40–49 years	21.7%	19.2%	20.4%	19.8%
	50–59 years	17.6%	12.4%	15.6%	14.0%
	60–74 years	22.9%	16.8%	17.6%	17.2%
	Total	100.0%	100.0%	100.0%	100.0%
Size of settlement	less than 1000 inhabitants	16.9%	9.3%	10.1%	9.7%
	1000 to 4999 inhabitants	22.3%	25.4%	22.6%	24.0%
	5000 to 19,999 inhabitants	18.0%	22.2%	23.8%	23.0%
	20,000 to 99,999 inhabitants	20.1%	17.4%	19.4%	18.4%
	100,000 inhabitants or more	22.7%	25.8%	24.2%	25.0%
	Total	100.0%	100.0%	100.0%	100.0%
Education	Elementary	14.0%	7.6%	9.2%	8.4%
	Vocational	32.9%	31.6%	28.4%	30.0%
	Secondary	34.5%	44.4%	44.4%	44.4%
	University	18.7%	16.4%	18.0%	17.2%
	Total	100.0%	100.0%	100.0%	100.0%

**Table 2:** Descriptive Statistics of the C2ST Scale and Its Items.

	N	Mean	SD	ITC	Alpha if Item Deleted
A. If I forget my device at home, I would go back to get it.	251	3.94	2.127	0.815	0.966
B. I sleep with my device on.	251	3.73	2.239	0.826	0.966
C. I spread the idea of self-tracking.	251	3.65	1.987	0.857	0.965
D. I share my data with others.	251	3.34	2.016	0.828	0.966
E. I make extra effort to reach my goal.	251	4.19	1.927	0.852	0.965
F. I recommend self-tracking to others.	251	3.88	1.982	0.846	0.965
G. I run an analysis of my tracked behavior (e.g., plot my running speed).	251	3.74	2.065	0.869	0.965
H. I am willing to pay extra for a “good quality” tracker.	251	3.96	1.961	0.831	0.966
I. I interact with a self-tracking community.	251	4.16	1.930	0.837	0.966
J. If someone beats me (in an online competition), I try to conquer my position again.	251	3.72	1.931	0.818	0.966
K. I purchase accessories for my tracker.	251	3.58	1.959	0.804	0.966
L. I update my pre-determined goals whenever required.	251	4.09	1.909	0.829	0.966

**Table 3:** Exploratory Factor Analysis (C2ST Scale).

	N	F1	Communalities
A. If I forget my device at home, I would go back to get it.	251	0.828	0.685
B. I sleep with my device on.	251	0.839	0.704
C. I spread the idea of self-tracking.	251	0.870	0.757
D. I share my data with others.	251	0.841	0.708
E. I make extra effort to reach my goal.	251	0.869	0.755
F. I recommend self-tracking to others.	251	0.861	0.741
G. I run an analysis of my tracked behavior (e.g., plot my running speed).	251	0.885	0.783
H. I am willing to pay extra for a “good quality” tracker.	251	0.846	0.715
I. I interact with a self-tracking community.	251	0.852	0.727
J. If someone beats me (in an online competition), I try to conquer my position again.	251	0.834	0.695
K. I purchase accessories for my tracker.	251	0.817	0.668
L. I update my pre-determined goals whenever required.	251	0.845	0.715
Eigenvalue		8.930	

**Table 4:** Absolute and Incremental Indices.

	Bollen–Stine *p*-Value	RMSEA	SRMR	GFI	CFI	TLI	NFI
Original model	*p* < 0.001	0.101	0.0284	0.881	0.957	0.947	0.941
Improved model	*p* = 0.173	0.050	0.0177	0.950	0.990	0.986	0.975

**Table 5:** Associations of C2ST with Other Indicators.

	%	Mean	SD	U/H	*p*-Value	*ε* ^2^ */δ*
Health status				21.477	<0.001 ^2^	0.08
good	78.4%	49.05	19.397			
neither, nor	16.4%	37.46	21.236			
bad	5.2%	28.38	13.956			
Physical status				2123.500	0.023 ^1^	−0.27
good	89.6%	47.24	19.975			
bad	10.4%	37.31	21.720			
Mental status				1916.000	0.138 ^1^	−0.20
good	90.8%	47.04	19.871			
bad	8.4%	39.24	24.205			
I want to compare my results with other people’s results.				96.894	<0.001 ^2^	0.39
agree	35.3%	61.97	13.089			
neither, nor	16.1%	48.98	16.599			
disagree	48.6%	34.26	17.361			
I want to show my data to others.				101.461	<0.001 ^2^	0.40
agree	33.3%	62.94	13.137			
neither, nor	14.9%	49.54	17.001			
disagree	51.8%	34.61	16.894			
I feel less anxious about my health.				33.715	<0.001 ^2^	0.13
agree	40.2%	55.7	18.257			
neither, nor	32.0%	43.62	16.693			
disagree	27.9%	38.22	21.027			
I consider tracking changes in my health as:				2813.500	<0.001 ^1^	−0.55
useful	70.5%	52.21	18.491			
not useful	29.5%	32.96	17.524			
I consider getting rid of a bad habit related to my health as:				3836.500	<0.001 ^1^	−0.48
useful	53.3%	54.19	17.849			
not useful	46.7%	37.44	18.818			
I consider motivating myself to achieve my health goals as:				3169.000	<0.001 ^1^	−0.56
useful	59.8%	54.50	17.230			
not useful	40.2%	34.53	18.142			
I consider tracking my progress in sports and training as:				2921.000	<0.001 ^1^	−0.59
useful	51.7%	56.96	15.648			
not useful	48.3%	35.70	18.816			
Frequency of use of the self-tracking device				45,138	<0.001 ^2^	0.17
several times a day/constantly	75.3%	52.86	18.558			
once a day	10.8%	35.20	18.642			
several times a week	10.8%	34.36	15.383			
less often	3.1%	32.55	20.680			

^1^ = Exact Mann–Whitney U; ^2^ = Kruskal–Wallis H; *ε*^2^ *=* epsilon squared (for Kruskal–Wallis test), *δ =* Cliff’s delta (for Mann–Whitney test).

## Data Availability

The data used to support the findings of this study will be available from the authors upon reasonable request.

## Abbreviations

The following abbreviations are used in this manuscript:

AVE	Average Variance Extracted
CFA	Confirmatory factor analysis
CFI	Comparative Fit Index
CR	Composite Reliability
C2ST	Commitment to self-tracking
DF	degrees of freedom
EFA	Exploratory factor analysis
GFI	goodness of fit index
ITC	item-total correlation
KMO	Kaiser–Meyer–Olkin
NFI	Normed Fit Index
PAF	Principal Axis Factoring
RMSEA	Root Mean Square Error of Approximation
SD	Standard deviation
SRMR	Standardized Root Mean Squared Residual
TLI	Tucker–Lewis Index

## Appendix A

**Table A1:** Correlation of C2ST Scale Items.

	Item A	Item B	Item C	Item D	Item E	Item F	Item G	Item H	Item I	Item J	Item K	Item L
Item A	1.000											
Item B	0.644 **	1.000										
Item C	0.626 **	0.698 **	1.000									
Item D	0.637 **	0.637 **	0.682 **	1.000								
Item E	0.579 **	0.558 **	0.598 **	0.593 **	1.000							
Item F	0.570 **	0.601 **	0.708 **	0.647 **	0.644 **	1.000						
Item G	0.581 **	0.642 **	0.653 **	0.648 **	0.678 **	0.682 **	1.000					
Item H	0.561 **	0.578 **	0.571 **	0.549 **	0.606 **	0.567 **	0.655 **	1.000				
Item I	0.559 **	0.565 **	0.575 **	0.606 **	0.660 **	0.626 **	0.619 **	0.628 **	1.000			
Item J	0.523 **	0.562 **	0.570 **	0.606 **	0.619 **	0.567 **	0.640 **	0.590 **	0.618 **	1.000		
Item K	0.594 **	0.562 **	0.595 **	0.575 **	0.549 **	0.580 **	0.581 **	0.651 **	0.557 **	0.584 **	1.000	
Item L	0.563 **	0.536 **	0.548 **	0.541 **	0.664 **	0.573 **	0.658 **	0.635 **	0.639 **	0.638 **	0.609 **	1.000

Note: Kendall’s tau_b; ** Correlation is significant at the 0.01 level; A. If I forget my device at home, I would go back to get it; B. I sleep with my device on; C. I spread the idea of self-tracking; D. I share my data with others; E. I make extra effort to reach my goal; F. I recommend self-tracking to others; G. I run an analysis of my tracked behavior (e.g., plot my running speed); H. I am willing to pay extra for a “good quality” tracker; I. I interact with a self-tracking community; J. If someone beats me (in an online competition), I try to conquer my position again; K. I purchase accessories for my tracker; L. I update my pre-determined goals whenever required.
